# Prevalence of MGCS Among Patients With Monoclonal Gammopathies

**DOI:** 10.1097/HS9.0000000000000908

**Published:** 2023-05-31

**Authors:** Foteini Theodorakakou, Despina Fotiou, Maria Gavriatopoulou, Ioannis Ntanasis-Stathopoulos, Vassiliki Spiliopoulou, Panagiotis Malandrakis, Magdalini Migkou, Evangelos Eleutherakis-Papaiakovou, Nikolaos Kanellias, Evangelos Terpos, Meletios A. Dimopoulos, Efstathios Kastritis

**Affiliations:** 1 Department of Clinical Therapeutics, Alexandra Hospital, National and Kapodistrian University of Athens, Greece

Monoclonal gammopathies is a common condition especially among elderly people,^1^ most commonly a “monoclonal gammopathy of undetermined significance” (MGUS), which usually remain asymptomatic, without characteristics of active malignancy.^2^ In some cases, monoclonal gammopathies are associated with a variety of syndromes that may involve the kidneys (characterized as monoclonal gammopathy of renal significance [MGRS]),^3^ peripheral nerve, skin, or less commonly organs such as the eyes, the muscles, or may even cause nonorgan-confined systemic syndromes. These syndromes have been termed as “monoclonal gammopathy of clinical significance” (MGCS),^4^ which usually are associated with a nonmalignant B-cell or plasma cell clones and do not fulfill the typical criteria of symptomatic multiple myeloma (MM) or Waldenström’s macroglobulinemia (WM).^5^ The manifestations of MGCS result from various properties of the monoclonal protein (deposition, autoantibody activity, or other mechanisms) or of the clone (such as ectopic production of cytokines), while in some syndromes the pathophysiological mechanisms remain unknown. Their diagnosis is challenging, requiring a high degree of suspicion or target organ biopsies. Because of the difficulties in their recognition, their true prevalence among patients with monoclonal gammopathies is unknown. On the contrary, a recent prospective study in Iceland reported a prevalence of monoclonal gammopathies of almost 5% among subjects aged >40 years.^6^ Thus, the prevalence of MGCS may be significantly underestimated due to the heterogeneous phenotype, the difficulties in establishing the diagnosis, and the low degree of suspicion.

To provide an estimate of the prevalence of MGCS among patients with monoclonal gammopathies, we analyzed the database of a referral center (Plasma Cell Dyscrasia Unit, Department of Clinical Therapeutics, Athens, Greece). This prospectively maintained database includes all patients with monoclonal gammopathies who are referred for evaluation or treatment. The analysis included all patients with monoclonal gammopathy with or without any concomitant symptoms or any laboratory abnormalities. Because of an increasing interest in MGCS after 2010, and subsequent rising awareness and diagnostic sensitivity, we excluded earlier referrals and focused only on 3138 consecutive patients who were referred and diagnosed with a monoclonal gammopathy between January 1, 2010 and December 31, 2021. The final diagnosis was a MGCS-related syndrome in 135 patients (4.3%) and was MGUS or smoldering myeloma (SMM) or asymptomatic WM (aWM) in 1437 patients (46%), symptomatic MM in 1145 patients (36%), symptomatic WM in 108 patients (3%), and immunoglobulin light chain (AL) amyloidosis in 320 patients (10%). In particular, the proportion of MGCS among those with <10% clonal cells was 5.8%, and among those with >10% clonal cells was 3.8%. Among those with MGCS, the majority was diagnosed either with MGRS (n=51) or an IgM-related neuropathy (n=58). Other specific MGCS diagnoses included POEMS (Polyneuropathy, Organomegaly, Endocrinopathy, M-protein, Skin changes) syndrome (n=13), Schnitzler’s syndrome (n=3), necrobiotic xanthogranuloma (n=3), or scleromyxedema (n=2), and other rare syndromes such as TEMPI (Telangiectasias, Elevated erythropoietin and erythrocytosis, Monoclonal gammopathy, Perinephric fluid collections, Intrapulmonary shunting) syndrome (n=2), IgM retinopathy (n=1), Clarkson’s syndrome (n=1), and crystalglobulinemia (n=1). Among patients with MGRS, the majority (n=43) had monoclonal immunoglοbulin deposition disease (MIDD) (light chain deposition disease [LCDD]:31, heavy chain deposition disease [HCDD]:2, H/LCDD:10), 4 patients had C3 nephropathy, 2 patients had fibrillary glomerulonephritis, 1 patient had membranous proliferative glomerulonephritis (MPGN), and 1 patient had immunotactoid glomerulonephritis (GN). Peripheral neuropathy with a concomitant monoclonal gammopathy was the most common coexisting condition among non-AL amyloidosis patients, with 73% having IgM and 37% a non-IgM paraproteinemia. A causal relationship between monoclonal gammopathy and neuropathy was established for 42% of those with non-IgM neuropathy and these patients mainly had POEMS syndrome. The median age of the MGCS patients was 64 years, not significantly different in clinical terms from that of patients with SMM/MGUS, AL amyloidosis, symptomatic MM, and symptomatic WM, which was 65, 65, 67 and 70 years, respectively (Table 1). The baseline level of monoclonal immunoglobulin among MGCS patients was low (median, 0.4 g/dL) and significantly lower than in patients with MGUS/SMM/aWM (median, 1.04 g/dL). The median bone marrow (BM) infiltration by clonal cells in patients with MGCS was 10%, and was 7% in the MGUS/SMM group, 15% in patients with AL amyloidosis, 50% in patients with symptomatic MM, and 40% for patients with symptomatic WM. Among patients with MGCS, 56% had kappa light chain and 42% had lambda light chain, while 2% were biclonal (shown in more details in Table 1). Among patients with MGCS, there were very few who progressed to symptomatic myeloma (1 with MIDD and 1 with Clarkson, none of which fulfilled myeloma criteria at the time of diagnosis) and 1 patient to symptomatic WM with anemia and high bone marrow infiltration. Although there were several patients with bone marrow infiltration >10%, none of them progressed to fully blown myeloma with hypercalcemia, renal insufficiency, anemia, bone lesions (CRAB) criteria. Most patients with MGCS received anticlonal therapy, mostly bortezomib-based regimens, immunomodulators (IMiDs), and anti-CD38 monoclonal antibody, in the more recent periods. In patients with B-cell clones, therapy was anti-CD20 based (rituximab). Nonclone targeting therapies were used in patients with specific syndromes such as Schnitzler’s syndrome (pefloxacin and anakinra) and Clarkson’s disease (immunoglobulin). A significant proportion of patients with IgM-related neuropathy received only supportive therapy.

**Table 1:** Baseline Characteristics of Patients Included in the Analysis

		N	Median Age, y	M-peak Baseline, g/dL	Median BM Infiltration (%)	IgA (%)	IgG (%)	IgM (%)	Kappa (%)	Lambda (%)
MGCS		135	64	0.4	10	9	36	19.5	51.5	24
	MGRS	51	54	0.2	15	12	41	2%	66	33
	POEMS	13	58	0	3	23	77	0	8	92
	IgM neuropathy	58	66.5	0,57	0	0	0	100	90	10
	Necrobiotic xanthogranuloma	3	71	1.5	12	0	100	0	50	50
	Schnitzler	3	47	0.5	0	0	0	100	100	0
	Scleromyxedema	2		0.4	5	0	100	0	50	50
	TEMPI	2		2.2	30	0	100	0	100	0
	Clarkson	1		2.2	30	0	100	0	100	0
	IgM retinopathy	1		0.9	8	0	0	100	100	0
	Cryocrystalglobulinemia	1	46	0.95	8	0	100	0	100	0
MGUS/SMM/aWM		1437	65	1.04	7	21	68	11	65	35
	MGUS	927	70	0.6	5	15	57	24	62	38
	SMM	474	65	1.4	15	26	69	0	66	34
	aWM	36	69	1.02	20	0	0	100	73	27
AL		320	65	<0.2	15	13	35	2	23	67
MM		1145	67	2.88	50	24	56	0.2	69	31
WM		108	70	2.3	40	0	0	100	77	23

AL = immunoglobulin light chain amyloidosis; aWM = asymptomatic Waldenstrom Macroglobulinemia; BM = bone marrow; MGCS = monoclonal gammopathy of clinical significance; MGRS = monoclonal gammopathy of renal significance; MGUS = monoclonal gammopathy of undetermined significance; MM = multiple myeloma; M-protein, skin changes; POEMS = polyneuropathy, organomegaly, endocrinopathy; SMM = smoldering myeloma; TEMPI = telangiectasias, elevated erythropoietin and erythrocytosis, monoclonal gammopathy, perinephric fluid collections, intrapulmonary shunting; WM = Waldenström’s macroglobulinemia.

To the best of our knowledge, this is the first study attempting to evaluate the prevalence of MGCS among patients with monoclonal gammopathies and found that among our patients it was almost 4%. In our study, the most common diagnoses were MGRS and IgM-related neuropathy; AL amyloidosis, although considered in the spectrum of MGRS and MGCS,^7^ is associated with a more aggressive clinical presentation and dismal prognosis requiring immediate treatment, being a unique entity. However, given the prevalence of monoclonal gammopathy in the general population and the heterogeneous clinical presentation of these syndromes, there is probably a significant number of patients with MGCS who remain undiagnosed. These observations are of clinical relevance because patients with MGCS, unlike patients with MGUS, require treatment and may suffer from complications associated with an otherwise nonmalignant condition. Nonetheless, the establishment of MGCS diagnosis requires a high degree of clinical suspicion due to their rarity and highly heterogeneous clinical presentation. Importantly, the demographic and clonal characteristics of most patients with MGCS do not differ from other symptomatic or asymptomatic monoclonal gammopathies making the diagnosis even more challenging, although some specific patterns may exist (eg, POEMS was almost always associated with lambda light chain^8^ [except in 1 case], while LCDD was more often associated with kappa light chain isotype^9^). Patients often experience unexplained and apparently unrelated symptoms and the collaboration of different specialties such as hematologists, nephrologists, dermatologists, cardiologists, neurologists is crucial; often the diagnosis may require biopsy of the target organ, especially when kidneys and skin are involved^10,11^ and an experienced pathologist plays a key role in the diagnosis of these disorders.

The causal link of a monoclonal gammopathy with a syndrome is often difficult to prove; on the contrary, new entities may be included among the MGCS spectrum in the future. This has an impact on treatment decisions, as in most cases these diseases are not life-threatening but unnecessary chemotherapy should also be avoided. Therapy of MGCS is based on the use of anticlonal regimens and depends on the origin of the clone (plasma cell, lymphocyte, or lymphoplasmacytic clones),^12^ similar to that used for AL amyloidosis, most often based on the bortezomib combinations or IMiD-based when neuropathy (as in POEMS) predominates, while the availability of anti-CD38 monoclonal antibodies expands treatment options.^13,14^ However, in some of these syndromes, management may be based on the nonclonal targeting therapies, or just on supportive therapy or immunosuppressive agents.^15–17^

The major limitation of our study is that it included patients from a referral center, which is probably associated with a selection bias; however, it is unlikely that the true prevalence of these syndromes can be evaluated in the general population. Thus, our estimations reflect our center’s MGCS frequency rather than the real incidence of MGCS in the population, which remain to be fully described. An approach could be to evaluate their prevalence within the context of a screening program, but this would require a more intensive use of diagnostic tools. Our results should be further validated in other referral centers and ideally within well-designed collaborative studies in order to improve the diagnosis and management of patients with MGCS. It is also important that physicians across different disciplines be informed and educated about these conditions so that patients be appropriately referred and diagnosed and treated timely and accordingly.

## AUTHOR CONTRIBUTIONS

FT analyzed and collected data, performed statistical analysis, and wrote the article. DF, MG, IN-S, VS, PM, MM, EE-P, NK, ET, and MAD analyzed and collected data and critically reviewed the article. EK designed the study, analyzed and collected data, performed statistical analysis, and wrote the article.

## DISCLOSURES

EK has received honoraria from Amgen, Janssen, Genesis Pharma, Takeda, and Prothena. MAD has received honoraria from Amgen, Celgene, Janssen, and Takeda. ET has received honoraria from Medtronic, Celgene, Janssen, and Amgen, and is an editor for HemaSphere. MG has received honoraria from Takeda, Amgen, and Novartis. All the other authors have no conflicts of interest to disclose.

## SOURCES OF FUNDING

The authors declare no sources of funding for this manuscript.
